# Identification of Novel Vacuolin-1 Analogues as Autophagy Inhibitors by Virtual Drug Screening and Chemical Synthesis

**DOI:** 10.3390/molecules22060891

**Published:** 2017-05-27

**Authors:** Chang Chen, Yingying Lu, Ho Ming Siu, Jintao Guan, Longchao Zhu, Shuang Zhang, Jianbo Yue, Liangren Zhang

**Affiliations:** 1 State Key Laboratory of Natural and Biomimetic Drugs, School of Pharmaceutical Sciences, Peking University, Beijing 100191, China; c.chang@bjmu.edu.cn (C.C.); zshbu_love@126.com (S.Z.); 2 City University of Hong Kong Shen Zhen Research Institute, Shenzhen 518057, China; superchristie@sina.com (Y.L.); kam.guan@hotmail.com (J.G.); longchao.zhu@outlook.com (L.Z.); 3 Department of Biomedical Sciences, City University of Hong Kong, Hong Kong, China; hmsiu9-c@my.cityu.edu.hk

**Keywords:** autophagy, vacuolin-1, LC3B-II, SAR, inhibitor

## Abstract

Autophagy is a fundamental cellular degradation process which is essential for cell homeostasis, and dysfunctional autophagy has been associated with a variety of human diseases, such as cancer. Several autophagy chemical modulators have been applied in a number of preclinical or clinical trials against these autophagy related diseases, especially cancer. Small molecule vacuolin-1 potently and reversibly inhibits both endosomal-lysosomal trafficking and autophagosome-lysosome fusion, yet the molecular mechanisms underlying vacuolin-1 mediated autophagy inhibition remain unknown. Here, we first performed the virtual drug screening and identified 14 vacuolin-1 analogues as autophagy inhibitors. Based on these virtual screening results, we further designed and synthesized 17 vacuolin-1 analogues, and found that 13 of them are autophagy inhibitors and a couple of them are as potent as vacuolin-1. In summary, these studies expanded the pool of useful autophagy inhibitors and reveal the structural-activity relationship of vacuolin-1 analogues, which is useful for future development of vacuolin-1 analogues with high potency and for identification of the molecular targets of vacuolin-1.

## 1. Introduction

Autophagy contains at least three types, including macroautophagy, microautophagy, and chaperone-mediated autophagy, and macroautophagy (hereafter referred as autophagy) is the most common and best studied form. Autophagy is initiated when misfolded proteins or damaged organelles are sequestered by double-membrane autophagosomes, and is completed when autophagosomes fuse with lysosomes to form autolysosomes, inside which the sequestered contents are degraded by lysosomal acidic hydrolases into amino acids or fatty acids and recycled back to cytosol [1,2]. Autophagy is essential to maintain the cellular homeostasis by preventing damaged and harmful substances from accumulating inside cells, and thus is involved in a wide variety of cellular processes, from cell proliferation, differentiation, apoptosis, development, ageing, to immunity [3,4,5]. The initiation, elongation, membrane closure, and maturation of autophagic process are tightly controlled by the complicated interplay among several core molecular machineries, including the Unc-51-like kinase (ULK) complex, the class III phosphatidylinositol 3-kinase (PtdIns3k) complex [6,7], and the two ubiquitin-like protein conjugation complexes, MAP1LC3B (microtubule associated protein 1 light chain 3B, herein referred simply as LC3B) and ATG12-ATG5-ATG16L complex (ATG, autophagy-related). The LC3B lipidation converts the cytosolic LC3B (LC3B-I) to the autophagic vesicle-associated form (LC3B-II). The lipidated LC3B-II showed a punctate staining pattern and had faster electrophoretic mobility compared with diffused LC3B-I, which have been commonly used to monitor the autophagic process.

Dysfunctional autophagy has been associated with a variety of human diseases, including neurodegenerative disorders, cardiovascular diseases, infection, and cancer [8,9,10]. On one hand, downregulated autophagy in normal tissue can lead to tumorigenesis due to the accumulation of misfolded proteins and damaged organelles inside cells. On the other hand, autophagy is upregulated in many kinds of cancers, which allow the cancer cells to meet the energy needs under the increased metabolic consumption and to survive in the hypoxic microenvironment [11,12]. Cancer cells also utilize autophagy to resist chemotherapy and radiation therapy, whereas inhibiting autophagy renders the cancer cells more sensitive to chemotherapy and radiation therapy [13,14]. Not surprisingly, a number of pre-clinical and clinical trials have been ongoing to evaluate the anti-cancer effects of chloroquine (CQ) or hydroxychloroquine (HCQ), the lysosomotropic agent that blocks the degradation of the products of autophagy by inhibiting lysosome function, in combination with chemotherapy or other agents [15]. Yet the therapeutic activity of CQ and HCQ seems to be partially due to the modulation of autophagy-unrelated mechanisms [16]. Therefore, compounds that regulate autophagy in a highly specific manner will certainly strengthen the clinical utility of this anti-cancer therapeutic paradigm.

Prompted by the fact that many available autophagy chemical modulators lack either potency or specificity [17], chemicals were developed for autophagy inhibition [18] and we set up assays to screen small molecules affecting autophagy. We found that the small chemical vacuolin-1, originally found to interfere with specific steps in membrane traffic, potently inhibited the fusion between autophagosomes and lysosomes, resulting in the accumulation of autophagosomes [19]. Vacuolin-1 treatment also blocked the fusion between endosomes and lysosomes, thus inhibiting general endosomal-lysosomal degradation. Moreover, we found that vacuolin-1 indirectly activates RAB5A to block autophagosome-lysosome fusion [20]. Recently, vacuolin-1 has also been shown to bind to PIKfyve in vitro, yet how vacuolin-1 regulates PIKfyve and whether it directly binds to PIKfyve in vivo remains unknown [21].

Since the molecular entities of vacuolin-1 remains to be identified, here we identified a number of vacuolin-1 structure analogues by combining virtual drug screening and chemical synthesis. By examining their effects on autophagy inhibition through western blot and confirming by fluorescence image-based assay, we studied the structure-activity relationship (SAR) of these analogues, and found that several vacuolin-1 analogues can potently inhibit autophagy.

## 2. Results and Discussion

### 2.1. Identification of Novel Vacuolin-1 Analogues by Virtual Drug Screening

**Figure 1:**
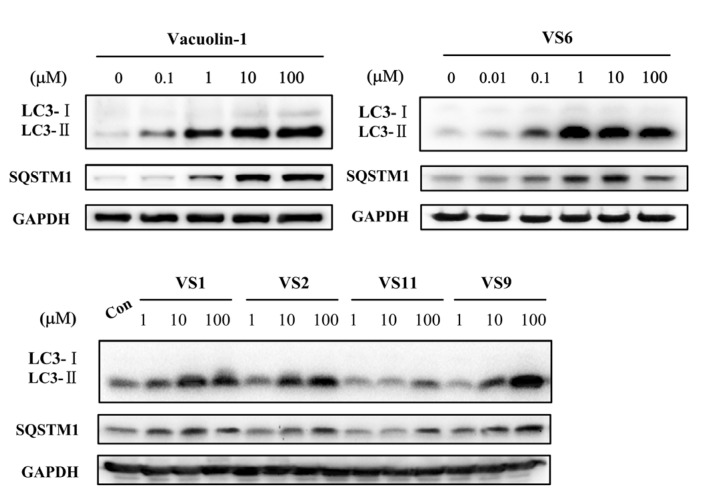
Vacuolin-1 analogues identified via virtual screening induced the accumulation of both LC3B-II and SQSTM1 in HeLa cells in a dose dependent manner after a 6 h treatment.

**Figure 2:**
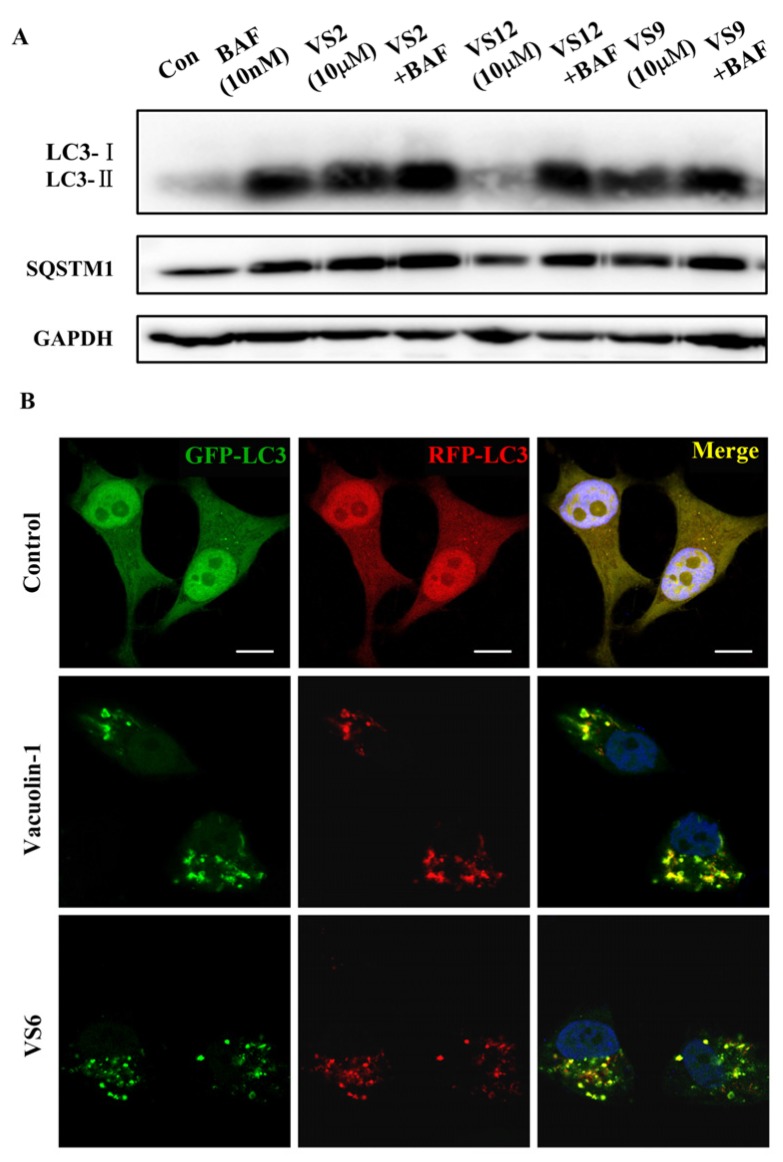
Vacuolin-1 analogues identified via virtual screening inhibited autophagic flux in HeLa cells. (**A**) Treatment of HeLa cells with vacuolin-1 analogues (10 µM) and BAF (100 nM) failed to further increase the accumulation of both LC3B-II and SQSTM1 as compared to either drug alone. (**B**) Vacuolin-1 (10 µM) or **VS6** (10 µM) induced the accumulation of yellow LC3B-II puncta in RFP-GFP-LC3B expressing HeLa cells. Scale bar = 10 µm.

**Figure 3:**
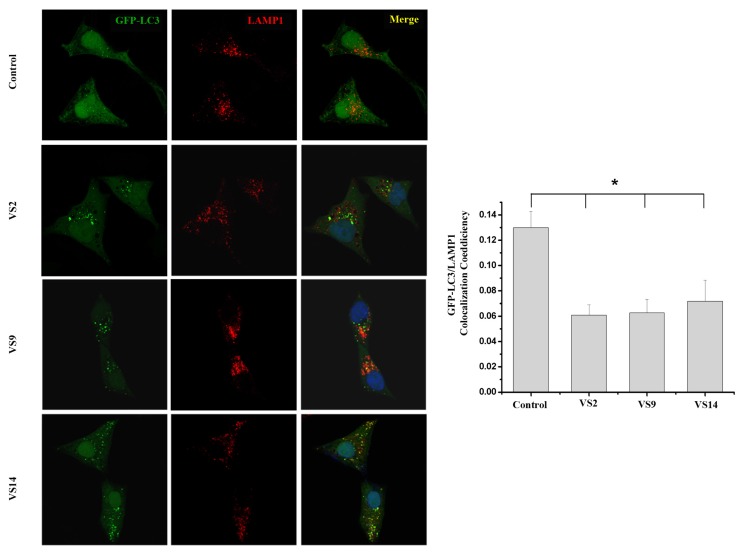
Vacuolin-1 analogues (10 µM) markedly induced GFP-LC3B-II puncta in HeLa cells, which were not colocalized with RFP-LAMP1. Scale bar = 10 µm. *, paired *t*-test, *p* < 0.05 compared to control group.

Since the molecular targets of vacuolin-1 remains unknown, we performed a 2D similarity search from ZINC database which contains over 13 million compounds and J & K Chemical database which contains more than 700,000 molecules by the Pipeline Pilot 7.5 software, and identified 14 vacuolin-1 analogues with the 1,3,5-triazine scaffold (Table 1). We subsequently analyzed the effects of these compounds on autophagy in tandem fluorescence-tagged LC3B (tfLC3B) expressing HeLa cells by both western blot and confocal image analyses. As shown in Figure 1 and Figure S1, except **VS11**, the rest of the analogues all induced the accumulation of both lapidated LC3B-II and SQSTM1, an autophagy substrate, in a dose dependent manner, albeit exhibiting different potency. In addition, co-treatment of cells with the vacuolin-1 analogues (10 µM) and bafilomycin (BAF) (100 nM), an inhibitor of the vacuolar proton pump that blocks the fusion of autophagosomes with lysosomes, failed to further induce the accumulation of LC3B-II and SQSTM1 as compared to either drug alone (Figure 2A). Likewise, treatment of cells with these vacuolin-1 analogues strongly induced the yellow LC3B-II puncta (representing the autophagosomes), not red-only LC3B-II puncta (representing autolysosomes) (Figure 2B and Figure S2). Moreover, the induced LC3B-II puncta did not colocalize with lysosome-associated membrane protein 1 (LAMP1) (Figure 3). Taken together, these data indicate that the identified vacuolin-1 analogues via drug virtual screening, like vacuolin-1, can potently inhibit the fusion between autophagosome and lysosomes, resulting in the accumulation of autophagosomes thus blocking autophagic flux. 

Comparing with vacuolin-1, **VS1**–**VS8** whose R^1^ groups were aromatic, maintained comparable autophagy inhibitory activity, whereas **VS9**–**VS14** which bearing two morpholino groups showed obviously decreased potency. The difference of **VS6** and **VS7** suggested that electron-withdrawing R^2^ substituted at the *meta*-position is more important for the autophagy inhibitory effects than *para*-position.

### 2.2. Identification of Novel Vacuolin-1 Analogues by Chemical Synthesis

**Scheme 1:**
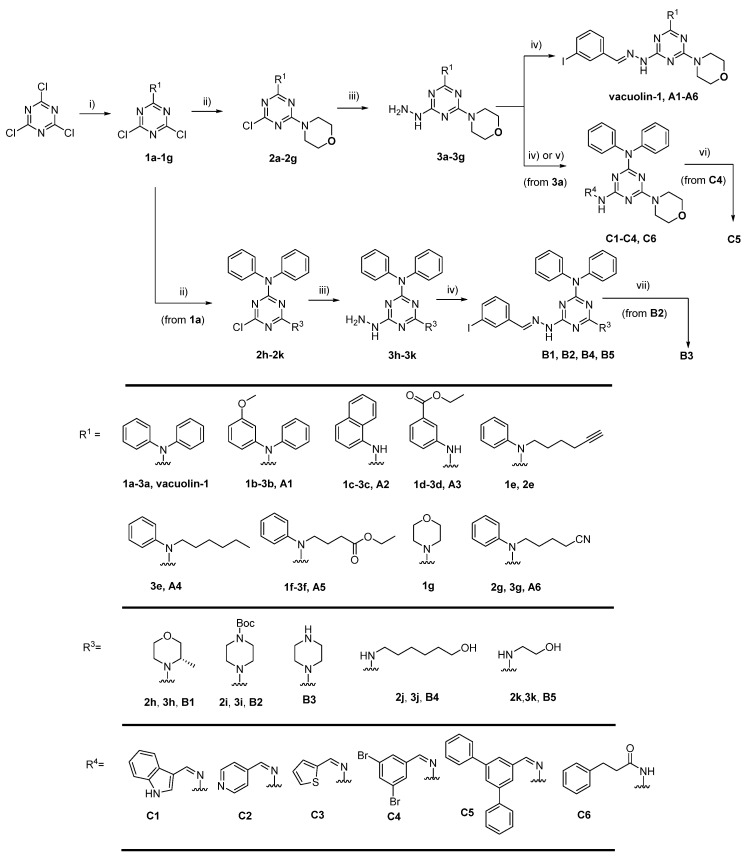
Reagents and conditions: (i) Amine, acetone, K_2_CO_3_, 0–5 °C →r.t, 65–94%; (ii) Amine, acetone, 0 °C →r.t, 67–86%. (for **1g**: K_2_CO_3;_ reflux; 42%); (iii) Dioxane, 80% hydrazine hydrate (m/m), r.t; (iv) Aldehyde, ethanol, CH_3_COOH, r.t, 55%–93%; (v) THF, acyl chloride, 0 °C →r.t, 73%; (vi) Phenylboronic acid, Pd(PPh_3_)_4_, Na_2_CO_3_, ethanol/ toluene/H_2_O (1:5:2, *v*/*v*), Ar, reflux, 12 h. 77%; (vii) DCM, TFA, r.t, 95%.

To further explore the SAR of vacuolin-1 on autophagy, we performed a systematic modification. The modifications could be divided into three parts based on the structure of vacuolin-1: the *N,N*-diphenylamino group modified analogues **A1**–**A6**, the morpholino group modified analogues **B1**–**B5,** and the aromatic moiety attached to hydrazine (henceforward hydrazine area) modified analogues **C1**–**C6**. The synthesis of vacuolin-1 analogues (Scheme 1) was carried out from trichlorotriazine to afford dichlorotriazine derivatives (**1a**–**1g**). Desired monoaddition products were obtained by controlling temperature and the excess amount of cyanuric chloride. Then **1a**–**1f** were converted to monochlorotriazines (**2a**–**2f** and **2h**–**2k**) after reacting with aliphatic amines with yields ranging from 67% to 86%. Compound **2g** was obtained from the reaction of **1g** with the corresponding aromatic amine under reflux due to low reactivity of the amine. Introduction of hydrazine on suitably derivatized monochlorotriazines provided **3a**–**3k**. Notably, the use of excess hydrazine hydrate converted the alkynyl group in **2e** to an alkyl group in **3e**. Condensation of **3a**–**3k** with 3-iodobenzaldehyde in the presence of acetic acid respectively provided vacuolin-1, **A1**–**A6**, **B1**, **B2**, **B4**, and **B5**. Deprotection of the Boc group in **B2** by TFA successfully produced **B3**. The reactions of intermediate **3a** with aldehydes gave **C1**–**C4**. Diphenyl substituent analogue **C5** was prepared from **C4** via Suzuki cross coupling with phenylboronic acid. Coupling of **3a** with hydrocinnamoyl chloride afforded target **C6**.

**Figure 4:**
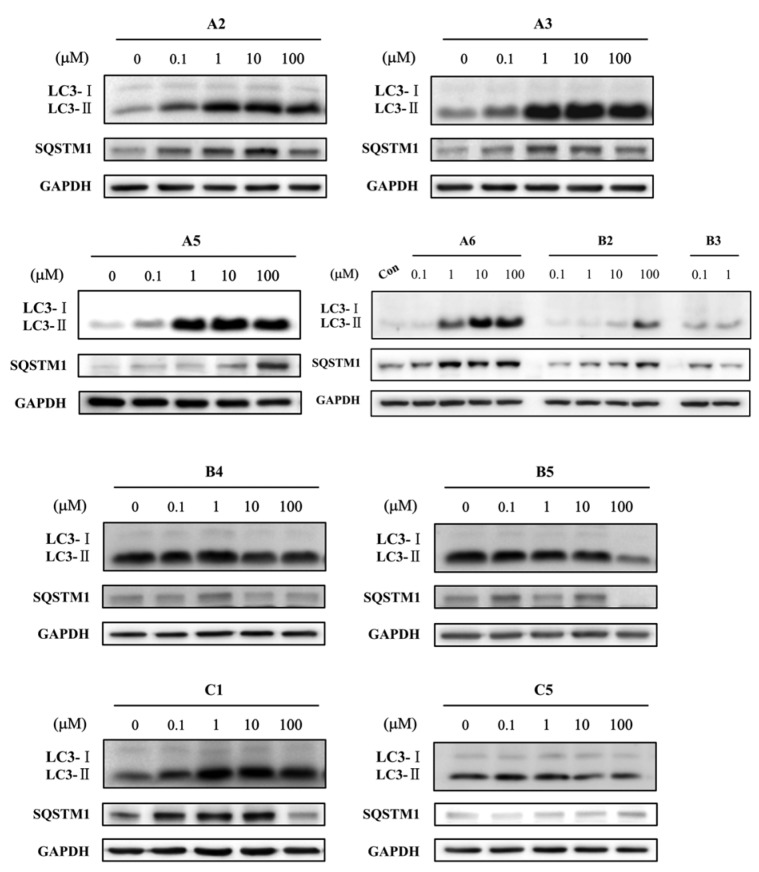
Synthesized vacuolin-1 analogues induced the accumulation of both LC3B-II and SQSTM1 in HeLa cells in a dose dependent manner after a 6 h treatment.

**Figure 5:**
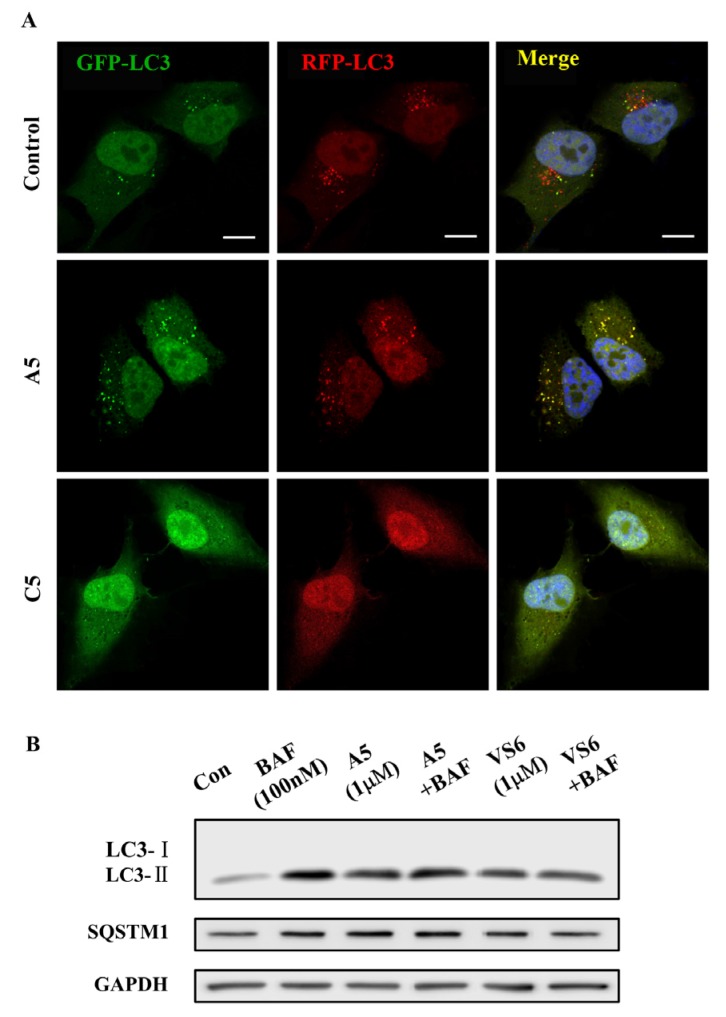
Synthesized vacuolin-1 analogues inhibited autophagic flux in HeLa cells. (**A**) **A5** (1 µM), not **C5** (1 µM), induced the accumulation of yellow LC3B-II puncta in RFP-GFP-LC3B expressing HeLa cells. Scale bar = 10 µm. (**B**) Treatment of HeLa cells with vacuolin-1 analogues (1 µM) and BAF (100 nM) failed to further increase the accumulation of both LC3B-II and SQSTM1 as compared to either drug alone.

Subsequently, we again analyzed the effects of these 17 vacuolin-1 analogues on autophagy in tfLC3B-expressing HeLa cells by both western blot and confocal image analyses. As shown in Figure 4, **S3**, and Table 2, analogues **B3**, **B4**, **B5**, and **C5** failed to induce the accumulation of both LC3B-II and SQSTM1 at all doses, analogues **B1**, **C2**, and **C6** only weakly induced the accumulation of LC3B-II and SQSTM1, analogues **A1**, **A2**, **A3**, **A6**, **C1**, and **C3** showed good potency, and analogues **A4** and **A5** were as potent as vacoulin-1. Similarly, the positive analogues, e.g., **A5** and others, markedly induced the yellow LC3B-II puncta in HeLa cells (Figure 5A and Figure S4), and co-treatment of cells with these analogues and bafilomycin (BAF) failed to further induce the accumulation of LC3B-II and SQSTM1 as compared to either drug alone (Figure 5B). Therefore, these data indicate that the synthesized vacuolin-1 analogues are autophagy inhibitors with good potency comparable to vacuolin-1.

**Figure 6:**
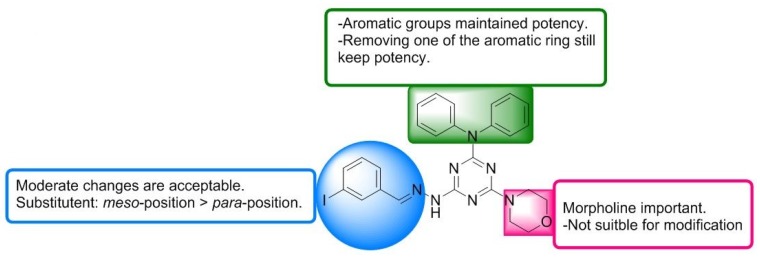
Structure-activity relationship for vacuolin-1 analogues as autophagy inhibitors.

Consistent with virtual screening results (Figure 1 and Table 1), removing one phenyl from diphenylamino group (**A2**–**A6**) maintained the ability to inhibit autophagy (Figure 4 and Table 2). The results not only suggested that aromatic R^1^ is important to keep autophagy inhibitory activity, but also implied that modification is tolerable on the phenyl group, particularly, analogues **A5** exhibited slightly stronger inhibitory potency than vacuolin-1 (Figure 4 and Table 2). Notably, small changes in morpholine caused a great loss of potency (**B1**–**B5**), indicating the crucial role of the morpholine ring in autophagy inhibition. As for the substituent in hydrazine area (**C1**–**C6**), moderate changes were acceptable, while bulky substituents killed the drug effects. The structure-activity relationship of vacuolin-1 analogues as autophagy inhibitors is summarized in Figure 6.

## 3. Experimental Section

### 3.1. 2D Similarity Virtual Screening

The research was performed on ZINC database and two collections of the J & K Chemical database (K66-X4436 EXPRESS-Pick Collection and L62-X6857 2013feb_sc1). Calculated by Pipeline Pilot 7.5 software (Accelrys, San Diego, CA, USA), the similarity of molecules and vacuolin-1 will be scored by Tanimoto coefficient described by FCFP_4 fingerprint. Molecules with Tanimoto great than 0.6 were screened out.

### 3.2. Chemistry

All of chemicals and solvents used were obtained from J & K or Sigma. The solvents were dried by standard procedures.^1^H NMR spectra and ^13^C NMR spectra were recorded at 400 MHz using Bruker Avance III spectrometer (Bruker BioSpin AG, Fällanden, Switzerland) in CDCl_3_ or DMSO-*d_6_* solution with tetramethylsilane as the internal standard and chemical shift values were given in ppm. The NMR data was processed by software MestReNova (Ver.6.1.0, Mestrelab research S.L., Santiago de Compostela, Spain). The high resolution mass (HRMS) was measured on an FT-MS-Bruker APEX IV mass spectrometer.

#### 3.2.1. General Procedure for the Synthesis of **1a**–**1g**

An amine (1 eq.) in acetone solution was added dropwise to a stirred solution of cyanuric chloride (1.5 eq.) and K_2_CO_3_ (1 eq.) in acetone at 0–5 °C. The resulting mixture was stirred at room temperature overnight. Ice water was added to the solution. The precipitate was collected by filtration and dried. The crude product was further purified by column chromatography.

#### 3.2.2. General Procedure for the Synthesis of **2a**–**2k**

General procedure for **2a**–**2f** and **2h**–**2k**: **1a**–**1g** or **2h**–**2k** (1 eq.), K_2_CO_3_ (1 eq.) were suspended in DCM, cooled to 0–5 °C with an ice bath while stirred. Amine solution (1.2 eq., 0.3 mol/L solution of DCM) was added dropwise. The resulting mixture was stirred at room temperature for an additional 30 min, monitored by TLC until the starting material disappeared. The solvent was evaporated and the residue was purified by column chromatography.

Procedure for **2g**: **1g** (1 eq.) was dissolved in acetone, K_2_CO_3_ (1 eq.) and amine (1.2 eq.) were added. The resulting mixture was stirred and refluxed overnight. The solvent was evaporated and partitioned between ethyl acetate and water. The organic phase was dried over with Na_2_SO_4_, filtered and concentrated in vacuo. The residue was purified by column chromatography.

#### 3.2.3. General Procedure for the Synthesis of **3a**–**3k**

**2a**–**2k** was dissolved in dioxane. Excess hydrazine hydrate (80%, m/m) was added to the solution while stirring. The reaction was monitored by TLC. After the staring material disappeared, water was added and then extracted using ethyl acetate (60 mL × 3). The combined organic phase was then washed with water, dried over with Na_2_SO_4_, filtered and concentrated in vacuo. The resulting solid (**3a**–**3k**) was used in the next condensing step without further purification.

#### 3.2.4. General Procedure for the Synthesis of Vacuolin-1, **A1**–**A6**, **B1**–**B5** and **C1**–**C6**

The reaction mixture under the conditions mentioned in Scheme 1 was stirred, and then evaporated to dryness under reduced pressure. The residue was purified by column chromatography.

*2-N,N-Diphenylamino-6-(2-(3-iodobenzylidene)hydrazinyl)-4-morpholino-1,3,5-triazine* (**vacuolin-1**). White solid. Mp 187.3–188.5 °C. ^1^H NMR (400 MHz, CDCl_3_) δ 8.59 (br s, 1H), 7.97 (s, 1H), 7.64 (d, *J* = 7.6 Hz, 2H), 7.54 (d, *J* = 6.4 Hz, 1H), 7.33 (dt, *J* = 15.2, 7.6 Hz, 8H), 7.21 (t, *J* = 7.0 Hz, 2H), 7.08 (t, *J* = 7.8 Hz, 1H), 3.69 (m, 8H). ^13^C NMR (101 MHz, CDCl_3_) δ 166.0, 165.2, 164.3, 143.8, 140.9, 138.2, 136.6, 135.5, 130.2, 128.7, 128.0, 126.2, 125.7, 94.5, 66.8, 43.6. HRMS (MS-ES^+^) *m*/*z* Calcd. for C_26_H_25_IN_7_O [M + H]^+^: 578.1165; found 578.1165.

*6-(2-(3-Iodobenzylidene)hydrazinyl)-2-(N-(3-methoxyphenyl)-N-phenylamino)-4-morpholino-1,3,5-triazine* (**A1**). White solid. Yield: 72%. Mp 187.3–188.5 °C. ^1^H NMR (400 MHz, DMSO-*d_6_*) δ 10.93 (s, 1H), 8.01 (s, 1H), 7.89 (s, 1H), 7.68 (d, *J* = 7.3 Hz, 1H), 7.54 (s, 1H), 7.38 (t, *J* = 7.1 Hz, 2H), 7.24 (m, 5H), 6.91 (s, 1H), 6.86–6.77 (m, 2H), 3.73 (s, 3H), 3.56 (s, 8H). ^13^C NMR (101 MHz, DMSO-*d_6_*) δ 166.2, 165.1, 164.7, 159.9, 145.4, 144.3, 138.0, 137.8, 131.2, 129.7, 129.1, 128.5, 126.1, 124.8, 120.7, 114.5, 111.6, 105.1, 95.6, 66.4, 55.7, 43.6. HRMS (MS-ES^+^) *m*/*z* Calcd. for C_27_H_27_IN_7_O_2_ [M + H]^+^: 608.1271; found 608.1271.

*6-(2-(3-Iodobenzylidene)hydrazinyl)-4-morpholino-2-(N-(naphthalen-1-yl)amino)-1,3,5-triazine* (**A2**). Light pink solid. Yield: 93%. Mp 225.5–226.4 °C. ^1^H NMR (400 MHz, DMSO-*d_6_*) δ 11.13 (s, 1H), 9.64 (s, 1H), 8.58 (br s, 1H), 8.17 (s, 1H), 8.14 (s, 1H), 7.93–7.79 (m, 4H), 7.74 (d, *J* = 7.4 Hz, 1H), 7.70 (d, *J* = 5.4 Hz, 1H), 7.49 (s, 1H), 7.36 (t, *J* = 7.2 Hz, 1H), 7.25 (t, *J* = 7.4 Hz, 1H), 3.82 (s, 4H), 3.70 (s, 4H). ^13^C NMR (101 MHz, DMSO-*d_6_*) δ 165.8, 164.8, 164.4, 140.3, 137.4, 137.4, 134.8, 134.2, 133.8, 130.8, 128.9, 128.0, 126.0, 125.8, 125.5, 125.5, 124.8, 123.5, 122.7, 95.2, 66.0, 43.2. HRMS (MS-ES^+^) *m*/*z* Calcd. for C_24_H_23_IN_7_O [M + H]^+^: 552.1009; found 552.1004.

*2-(N-(3-Ethoxycarbonyl)phenylamino-6-(2-(3-iodobenzylidene)hydrazinyl)-4-morpholino-1,3,5-triazine* (**A3**). White solid. Yield: 75%. Mp 194.9–195.3 °C. ^1^H NMR (400 MHz, CDCl_3_) δ 8.99 (s, 1H), 8.47 (s, 1H), 8.02 (s, 1H), 7.76–7.57 (m, 4H), 7.51 (d, *J* = 6.9 Hz, 1H), 7.34 (t, *J* = 7.5 Hz, 1H), 7.05 (t, *J* = 7.7 Hz, 1H), 4.36 (q, *J* = 7.0 Hz, 2H), 3.91 (s, 4H), 3.78 (s, 4H), 1.38 (t, *J* = 7.0 Hz, 3H). ^13^C NMR (101 MHz, CDCl_3_) δ 166.5, 165.2, 164.5, 164.3, 141.4, 139.4, 138.5, 136.1, 135.6, 131.0, 130.2, 128.8, 126.6, 124.2, 124.0, 121.2, 94.6, 66.9, 61.1, 44.1, 14.5. HRMS (MS-ES^+^) *m*/*z* Calcd. for C_23_H_25_IN_7_O_3_ [M + H]^+^: 574.1064; found 574.1085.

*2-N-Hexyl-N-phenylamino-6-(2-(3-iodobenzylidene)hydrazinyl)-4-morpholino-1,3,5-triazine* (**A4**). White solid. Yield: 72%. Mp 140.3–141.5 °C. ^1^H NMR (400 MHz, CDCl_3_) δ 8.38 (s, 1H), 7.98 (s, 1H), 7.64 (m, 2H), 7.55 (d, *J* = 6.7 Hz, 1H), 7.39 (t, *J* = 7.4 Hz, 2H), 7.27 (s, 2H), 7.07 (t, *J* = 7.7 Hz, 1H), 3.95 (t, *J* = 8.0 Hz, 2H), 3.90–3.55 (m, 8H), 1.63 (t, *J* = 6.4 Hz, 2H), 1.35–1.24 (m, 6H), 0.86 (t, *J* = 5.5 Hz, 2H). ^13^C NMR (101 MHz, CDCl_3_) δ 165.5, 165.2, 164.2, 143.4, 140.4, 140.4, 138.1, 136.8, 135.5, 130.1, 128.7, 127.9, 126.1, 94.4, 66.9, 49.9, 43.7, 31.6, 28.0, 26.6, 22.6, 14.0. HRMS (MS-ES^+^) *m*/*z* Calcd. for C_26_H_33_IN_7_O [M + H]^+^: 586.1791; found 586.1796.

*2-(N-(4-Ethoxy-4-oxobutyl)-N-phenylamino)-6-(2-(3-iodobenzylidene)hydrazinyl)-4-morpholino-1,3,5-triazine* (**A5**). White solid. Yield: 92%. Mp 146.5–147.8 °C. ^1^H NMR (400 MHz, CDCl_3_) δ 8.53 (s, 1H), 7.99 (s, 1H), 7.65 (d, *J* = 7.8 Hz, 2H), 7.57 (d, *J* = 4.0 Hz, 1H), 7.40 (t, *J* = 7.7 Hz, 2H), 7.28 (dd, *J* = 9.5, 5.6 Hz, 3H), 7.09 (t, *J* = 7.8 Hz, 1H), 4.11 (m, 2H), 4.05 (t, *J* = 7.1 Hz, 2H), 3.78 (br s, 8H), 2.38 (t, *J* = 7.4 Hz, 2H), 2.04–1.94 (m, 2H), 1.23 (t, *J* = 7.1 Hz, 3H). ^13^C NMR (101 MHz, CDCl_3_) δ 173.2, 165.6, 165.2, 164.2, 143.1, 140.5, 138.1, 136.7, 135.5, 130.2, 128.9, 127.9, 126.3, 126.1, 94.4, 66.9, 60.4, 49.1, 43.7, 31.7, 23.5, 14.3. HRMS (MS-ES^+^) *m*/*z* Calcd. for C_26_H_31_IN_7_O_3_ [M + H]^+^: 616.1533; found 616.1526.

*2-(N-(4-Cyanobutyl)-N-phenylamino)-6-(2-(3-iodobenzylidene)hydrazinyl)-4-morpholino-1,3,5-triazine* (**A6**). White solid. Yield: 84%. Mp 112.5–113.8 °C ^1^H NMR (400 MHz, CDCl_3_) δ 8.98 (s, 1H), 7.95 (s, 1H), 7.66–7.48 (m, 3H), 7.35 (t, *J* = 6.2 Hz, 2H), 7.27–7.17 (m, 3H), 7.05 (t, *J* = 8.0 Hz, 1H), 4.04 (s, 2H), 3.85–3.53 (m, 8H), 2.44 (s, 2H), 1.77 (d, *J* = 4.7 Hz, 2H), 1.70 (d, *J* = 4.9 Hz, 2H). ^13^C NMR (101 MHz, CDCl_3_) δ 165.5, 164.9, 164.0, 142.6, 140.5, 137.8, 136.5, 135.2, 130.1, 128.7, 127.7, 126.2, 125.9, 119.5, 94.3, 66.5, 48.0, 43.5, 26.8, 22.3, 16.6. HRMS (MS-ES^+^) *m*/*z* Calcd. for C_25_H_28_IN_8_O [M + H]^+^: 583.1431; found 583.1447.

*(S)-2-N,N-Diphenylamino-6-(2-(3-iodobenzylidene)hydrazinyl)-4-(3-methylmorpholino)-1,3,5-triazine* (**B1**). White solid. Yield 70%. Mp 219.6–210.5 °C. ^1^H NMR (400 MHz, CDCl_3_) δ 9.09 (br s, 1H), 7.96 (s, 1H), 7.63 (d, *J* = 7.8 Hz, 1H), 7.54 (d, *J* = 6.2 Hz, 2H), 7.34 (m, *J* = 7.9 Hz, 8H), 7.20 (t, *J* = 6.5 Hz, 2H), 7.07 (t, *J* = 7.8 Hz, 1H), 4.94–2.99 (m, 7H), 1.23 (s, 3H). ^13^C NMR (101 MHz, CDCl_3_) δ 165.9, 164.9, 164.2, 143.8, 141.0, 138.1, 136.7, 135.5, 130.2, 128.7, 127.9, 126.1, 125.7, 94.6, 71.0, 67.0, 46.3, 38.6, 14.5. HRMS (MS-ES^+^) *m*/*z* Calcd. for C_27_H_27_IN_7_O [M + H]^+^: 592.1322; found 592.1323.

*4-(4-(t-Butyloxycarbonyl)piperazin-1-yl)-2-N,N-diphenylamino-6-(2-(3-iodobenzylidene)hydrazinyl)-1,3,5-triazine* (**B2**). White solid. Yield 83%. Mp 238.9–240.3 °C. ^1^H NMR (400 MHz, CDCl_3_) δ 8.36 (br s, 1H), 8.00 (s, 1H), 7.71 (s, 1H), 7.67 (d, *J* = 7.7 Hz, 1H), 7.56 (s, 1H), 7.41–7.31 (m, 8H), 7.24 (t, *J* = 7.1 Hz, 2H), 7.11 (t, *J* = 7.8 Hz, 1H), 3.87 (s, 2H), 3.51 (s, 4H), 3.39 (s, 2H), 1.49 (s, 9H). ^13^C NMR (101 MHz, CDCl_3_) δ 166.1, 165.2, 164.3, 154.8, 143.8, 140.8, 138.2, 135.5, 130.2, 128.7, 128.0, 126.2, 125.7, 94.5, 80.0, 43.0, 28.4. HRMS (MS-ES^+^) *m*/*z* Calcd. for C_31_H_34_IN_8_O_2_ [M + H]^+^: 677.1849; found 677.1865.

*2-N,N-Diphenylamino-6-(2-(3-iodobenzylidene)hydrazinyl)-4-(piperazin-1-yl)-1,3,5-triazine* (**B3**). White solid. Yield 90%. Mp 210.9–211.5 °C. ^1^H NMR (400 MHz, DMSO-*d_6_*) δ 11.09 (s, 1H), 8.95 (s, 2H), 8.02 (s, 1H), 7.92 (s, 1H), 7.71 (d, *J* = 7.9 Hz, 1H), 7.43–7.28 (m, 8H), 7.25 (t, *J* = 6.6 Hz, 2H), 7.19 (t, *J* = 7.9 Hz, 1H), 4.11–3.47 (m, 4H), 3.12 (s, 4H). ^13^C NMR (101 MHz, DMSO-*d_6_*) δ 166.2, 165.0, 164.7, 158.7, 144.2, 141.3, 138.0, 137.8, 134.8, 131.3, 129.2, 128.5, 126.2, 95.6, 42.8. HRMS (MS-ES^+^) *m*/*z* Calcd. for C_26_H_26_IN_8_O [M + H]^+^: 577.1325; found 577.1320.

*2-N,N-Diphenylamino-4-((6-hydroxyhexyl)amino)-6-(2-(3-iodobenzylidene)hydrazinyl)-1,3,5-triazine* (**B4**). White solid. Yield 60%. Mp 87.3–88.0 °C. ^1^H NMR (400 MHz, DMSO-*d_6_*) δ 10.85 (m, 1H), 7.98 (m, 2H), 7.68 (d, *J* = 6.7 Hz, 1H), 7.49 (d, *J* = 6.8 Hz, 1H), 7.35 (d, *J* = 7.2 Hz, 4H), 7.30 (d, *J* = 6.8 Hz, 4H), 7.22–7.14 (m, 3H), 4.35 (s, 1H), 3.40–3.36 (m, 2H), 2.94 (d, *J* = 5.7 Hz, 1H), 1.58–0.77 (m, 10H). ^13^C NMR (101 MHz, DMSO-*d_6_*) δ 166.4, 166.2, 164.5, 144.6, 140.1, 138.0, 137.7, 134.1, 131.2, 129.0, 128.6, 126.8, 125.8, 95.7, 61.2, 33.1, 29.7, 26.8, 25.7, 19.4. HRMS (MS-ES^+^) *m*/*z* Calcd. for C_28_H_31_IN_7_O [M + H]^+^: 608.1635; found 608.1649.

*2-N,N-Diphenylamino-4-((2-hydroxyethyl)amino)-6-(2-(3-iodobenzylidene)hydrazinyl)-1,3,5-triazine* (**B5**). White solid. Yield 55%. Mp 229.5–231.0 °C. ^1^H NMR (400 MHz, DMSO-*d_6_*) δ 11.01 (s, 1H), 8.00 (m, 2H), 7.69 (d, *J* = 7.6 Hz, 1H), 7.50 (d, *J* = 6.3 Hz, 1H), 7.37 (t, *J* = 7.6 Hz, 4H), 7.29 (d, *J* = 7.7 Hz, 4H), 7.25–7.15 (m, 3H), 4.49 (t, *J* = 4.8 Hz, 1H), 3.52 (s, 1H), 3.33 (m, 2H), 3.05 (dd, *J* = 11.5, 5.8 Hz, 1H). ^13^C NMR (101 MHz, DMSO-*d_6_*) δ 166.5, 166.3, 164.5, 144.7, 144.5, 140.8, 138.0, 134.2, 131.3, 131.2, 129.3, 129.1, 128.8, 128.6, 126.8, 126.0, 125.9, 95.7, 60.3, 43.3. HRMS (MS-ES^+^) *m*/*z* Calcd. for C_24_H_23_IN_7_O [M + H]^+^: 552.1009; found 552.1011.

*2-N,N-Diphenylamino-6-(2-((1H-indol-3-yl)methylene)hydrazinyl)-4-morpholino-1,3,5-triazine* (**C1**). White solid. Yield 93%. Mp 273.6–274.9 °C. ^1^H NMR (400 MHz, DMSO-*d_6_*) δ 10.57 (s, 1H), 8.24 (s, 1H), 7.71 (d, *J* = 6.7 Hz, 1H), 7.61 (s, 1H), 7.45–7.29 (m, 8H), 7.21–7.10 (m, 2H), 6.90 (t, *J* = 6.9 Hz, 1H), 6.72 (s, 2H), 6.47 (d, *J* = 7.9 Hz, 1H), 6.34 (t, *J* = 7.3 Hz, 1H), 3.69–3.39 (m, 8H). ^13^C NMR (101 MHz, DMSO-*d_6_*) δ 166.5, 165.0, 164.5, 144.7, 140.0, 139.9, 137.4, 129.0, 128.6, 125.8, 124.7, 123.0, 122.8, 120.6, 113.0, 111.9, 66.4, 43.6. HRMS (MS-ES^+^) *m*/*z* Calcd. for C_28_H_27_N_8_O [M + H]^+^: 491.2308; found 491.2306.

*2-N,N-Diphenylamino-4-morpholino-6-(2-(pyridin-4-ylmethylene)hydrazinyl)-1,3,5-triazine* (**C2**). White solid. Yield 70%. Mp 262.3–263.7 °C. ^1^H NMR (400 MHz, CDCl_3_) δ 9.03 (s, 1H), 8.60 (d, *J* = 5.4 Hz, 2H), 7.74 (s, 1H), 7.48 (d, *J* = 4.6 Hz, 2H), 7.42–7.30 (m, 8H), 7.23 (t, *J* = 7.1 Hz, 2H), 4.03–3.47 (m, 8H). ^13^C NMR (101 MHz, CDCl_3_) δ 166.0, 165.1, 164.3, 150.1, 143.8, 142.0, 139.6, 128.7, 128.0, 125.8, 120.8, 66.8, 43.6. HRMS (MS-ES^+^) *m*/*z* Calcd. for C_25_H_25_N_8_O [M + H]^+^: 453.2151; found 453.2143.

*2-N,N-Diphenylamino-4-morpholino-6-(2-(thiophen-2-ylmethylene)hydrazinyl)-1,3,5-triazine* (**C3**). White solid. Yield 73%. Mp 242.8–243.5 °C. ^1^H NMR (400 MHz, DMSO-*d_6_*) δ 10.80 (s, 1H), 8.29 (s, 1H), 7.53 (d, *J* = 4.8 Hz, 1H), 7.36 (t, *J* = 7.5 Hz, 4H), 7.29 (d, *J* = 7.5 Hz, 4H), 7.23–7.16 (m, 3H), 7.10–7.04 (m, 1H), 3.97–3.44 (m, 8H). ^13^C NMR (101 MHz, DMSO-*d_6_*) δ 166.3, 165.01, 164.5, 144.5, 140.7, 138.1, 129.1, 129.0, 128.5, 128.1, 127.9, 125.9, 66.4, 43.6. HRMS (MS-ES^+^) *m*/*z* Calcd. for C_24_H_24_N_7_OS [M + H]^+^: 458.1763; found 458.1765.

*6-(2-(3,5-Dibromobenzylidene)hydrazinyl)-2-N,N-diphenylamino-4-morpholino-1,3,5-triazine* (**C4**). White solid. Yield 72%. Mp 225.4–226.7 °C. ^1^H NMR (400 MHz, CDCl_3_) δ 8.64 (br s, 1H), 7.65 (s, 2H), 7.59 (s, 1H), 7.39–7.28 (m, 8H), 7.22 (t, *J* = 6.9 Hz, 2H), 3.98–3.42 (m, 8H). ^13^C NMR (101 MHz, CDCl_3_) δ 166.0, 165.1, 164.3, 143.7, 139.4, 138.2, 134.4, 128.7, 128.3, 127.9, 125.8, 123.1, 66.8, 43.6. HRMS (MS-ES^+^) *m*/*z* Calcd. for C_26_H_24_Br_2_N_7_O [M + H]^+^: 608.0409; found 608.0410.

*2-N,N-Diphenylamino-4-morpholino-6-(2-((1,1':3',1''-terphenyl)-5'-ylmethylene)hydrazinyl)-1,3,5-triazine* (**C5**). White solid. Yield 77%. Mp 225.4–226.7 °C. ^1^H NMR (400 MHz, DMSO-*d_6_*) δ 11.05 (s, 1H), 8.24 (s, 1H), 7.86 (s, 1H), 7.82–7.74 (m, 5H), 7.54 (t, *J* = 7.0 Hz, 4H), 7.44 (t, *J* = 7.1 Hz, 2H), 7.39–7.28 (m, 8H), 7.17 (t, *J* = 5.7 Hz, 2H), 3.83–3.36 (m, 8H). ^13^C NMR (101 MHz, DMSO-*d_6_*) δ 165.0, 164.8, 144.5, 142.2, 141.8, 140.2, 137.0, 129.4, 129.0, 128.5, 128.3, 127.4, 126.3, 125.9, 124.1, 66.3, 43.6. HRMS (MS-ES^+^) *m*/*z* Calcd. for C_38_H_34_N_7_O [M + H]^+^: 604.2825; found 604.2826.

*2-N,N-Diphenylamino-4-morpholino-6-(2-(3-phenylpropanoyl)hydrazinyl)-1,3,5-triazine* (**C6**). White solid. Yield 73%. Mp 174.6–175.5 °C. ^1^H NMR (400 MHz, CDCl_3_) δ 8.16 (br s, 1H), 7.34–7.27 (m, 7H), 7.23–7.14 (m, 8H), 6.61 (br s, 1H), 3.78–3.47 (m, 8H), 2.96–2.79 (m, 2H), 2.51–2.12 (m, 2H). ^13^C NMR (101 MHz, CDCl_3_) δ 178.2, 167.5, 166.0, 164.9, 143.8, 140.7, 128.6, 128.3, 128.0, 127.6, 126.3, 125.6, 124.9, 66.7, 43.5, 35.8, 31.2. HRMS (MS-ES^+^) *m*/*z* Calcd. for C_28_H_30_N_7_O_2_ [M + H]^+^: 496.2461; found 496.2462.

### 3.3. Autophagy Inhibitory Activity Measurement

*Cell culture*—HeLa cells were maintained in DMEM (Invitrogen, 12800-017) plus 10% fetal bovine serum and penicillin/streptomycin at 5% CO_2_ and 37 °C.

*Antibodies and reagents*—LC3B (Novus, Littleton, CO, USA, NB100-2220; 1:1000 for the Western blotting analysis (WB)), SQSTM1 (Novus, NBP1-48320; 1:1000 WB), EGFR (Santa Cruz Biotechnology, Santa Cruz, CA, USA, SC-03; 1:250 WB), LAMP1 (Cell Signaling Technology, 9091; 1:500 immunofluorescence analysis), GAPDH antibodies (Sigma, Saint Louis, MO, USA, G8795; 1:5000 WB). BAF (Sigma-Aldrich, B1793), and Vacuolin-1 (Santa Cruz Biotechnology, SC-216045).

*GFP-LC3B and RFP-GFP-LC3B Lentivirus Production and Infection*—GFP-LC3B and RFP-GFP-LC3B (tfLC3B) cDNA was cloned into pLenti-CMV-Puro-DEST (Addgene). The lentivirus production was performed in 293T cells. For infection, cells were plated at a density of 3 × 105 cells/well in 6-well plates. The experiment was performed as described previously [20].

*Western blot analysis*—HeLa cells were treated with or without indicated vacuolin-1 analogues for 6h before lyzed by EBC lysis buffer (50 mM HEPES at pH 7.5, 0.15 M NaCl, 1 mM EDTA, 1% (m/m) NP-40, 150 μM PMSF, 10 mM NaF, 10 ng/mL leupeptin, 1 mM DTT, and 1 mM sodium vanadate). Protein concentrations of the cell lysates were determined by Bradford protein assay. 30 or 40 µg of cell lysates per lane were subjected to electrophoresis on 8%, 10%, or 12% SDS polyacrylamide gels, transferred to PVDF membrane, blocked with 5% (m/m) milk in TBST, and incubated with the primary antibody (anti-LC3B, Novus Biologicals; anti-SQSTM1, Novus Biologicals; anti-GAPDH, Sigma-Aldrich). After washing with TBST three times, the blots were probed with a secondary antibody (1:5000 dilution) for detection by chemiluminescence.

*Confocal imanging analysis*—RFP-GFP-LC3B or GFP-LC3B/RFP-Lamp1 expressing HeLa cells were treated with the indicated drugs for 6 h. Images were then captured under a Zeiss LSM710 confocal microscope with a Plan-Apochromot × 63/1.40 oil DIC objective. Images were processed and analyzed with Zeiss ZEN software (Zeiss, Göttingen, Germany).

## 4. Conclusions

Here we identified a number of novel vacuolin-1 analogues by combining virtual drug screening and chemical synthesis, and found that several of these analogues, e.g., **VS6**, **A4**, and **A5**, are as potent as vacuolin-1 in inhibiting autophagy (Table 1 and Table 2). Therefore, our study expanded the pool of useful autophagy inhibitors, which could be applied to dissect the mechanism of autophagy or endosomal trafficking and be used as potential therapeutic agents in autophagy related diseases, e.g., cancer. In addition, the SAR profile of vacuolin-1 analogues as autophagy inhibitors provided by the current study (Figure 6) could aid us to synthesize photoaffinity analogues or analogues with an alkyne tag for identifying its intracellular molecular targets.

## Figures and Tables

**Table 1:**
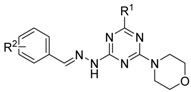
Structure-activity relationship (SAR) of selected compounds from virtual screening ^a^.

Compound	R^1^	R^2^	0.1 µM	1 µM	10 µM	100 µM
**Vacuolin-1**	*N,N*-diphenylamino	3-I	+	++	+++	++++
**VS1**	*N,N*-diphenylamino	4-F		+	+	+
**VS2**	4-Cl-phenylamino	4-Cl		−	+	+
**VS3**	4-Cl-phenylamino	3-I	+	++	++	++
**VS4**	4-F-phenylamino	3-I	+	++	++	++
**VS5**	4-Cl-phenylamino	4-dimethylamino	+	++	++	++
**VS6**	4-Cl-phenylamino	3-NO_2_	+	+++	+++	+++
**VS7**	4-Cl-phenylamino	4-NO_2_	−	−	+	+
**VS8**	4-OH-phenylamino	2,4-dimethyl	+	++	++	++
**VS9**	morpholino	H		−	+	+
**VS10**	morpholino	4-Cl		−	−	+
**VS11**	morpholino	4-Br		−	−	−
**VS12**	morpholino	4-I		−	−	+
**VS13**	morpholino	3,4-di-Cl		−	−	+
**VS14**	morpholino	3-Br-6-OMe		−	+	+

^a^ $\lambda =\frac{LC3-II\;Intensity}{GAPDH\;Intensity}$ . λ_0_: control group. “−”: λ_0_ ≤ 1.1; “+”: 1.1 < λ/λ_0_ ≤ 3; “++”: 3 < λ/λ_0_ ≤ 7; “+++”: 7 < λ/λ_0_ ≤ 10; “++++”: 10 < λ/λ_0_.

**Table 2:** SAR of synthesized compounds ^a^.

Compound	0.1 µM	1 µM	10 µM	100 µM	Compound	0.1 µM	1 µM	10 µM	100 µM
**vacuolin-1**	+	++	+++	++++	**B3**	−	−	*	
**A1**	+	++	++	++	**B4**	−	−	−	−
**A2**	+	++	++	++	**B5**	−	−	−	−
**A3**	+	++	++	++	**C1**	+	+	++	++
**A4**	+	+	+++	+++	**C2**	−	−	−	+
**A5**	+	+++	++++	++++	**C3**	−	−	++	++
**A6**	+	+	++	++	**C4**	−	−	−	+
**B1**	−	+	+	+	**C5**	−	−	−	−
**B2**	−	−	+	+	**C6**	−	−	−	++

^a^ $\;\lambda =\frac{LC3-II\;Intensity}{GAPDH\;Intensity}$ . λ_0_: control group. “−”: λ/λ_0_ ≤ 1.1; “+”: 1.1 < λ/λ_0_ ≤ 3; “++”: 3 < λ/λ_0_ ≤ 7; “+++”: 7 < λ/λ_0_ ≤ 10; “++++”: 10 < λ/λ_0_. * Toxic, 10 µM caused cell death.

## Supplementary Materials

Supplementary materials are available online.
