# Supplementary material for: Identification of Novel Vacuolin-1 Analogues as Autophagy Inhibitors by Virtual Drug Screening and Chemical Synthesis
Source: Molecules. 2017 May 27;22(6):891. doi: 10.3390/molecules22060891 (PMC6152680; doi:10.3390/molecules22060891)
Supplement: Supplementary file 1 [file molecules-22-00891-s001.pdf]

Supplementary information

Figure S1. Vacuolin-1 analogues identified via virtual screening induced the accumulation of both LC3B-II and SQSTM1 in HeLa cells in a dose dependent manner after 6 h treatment.

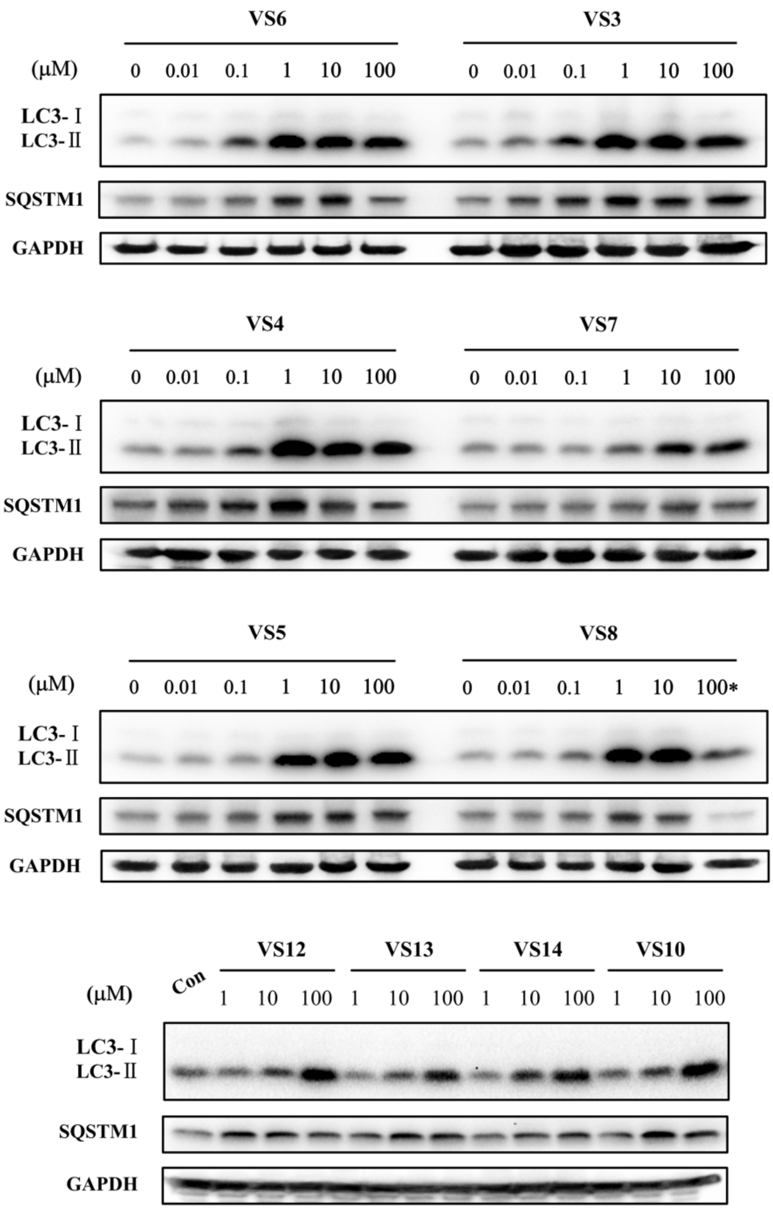

Figure S2. VS1 (10 μM), **VS9** (10 μM), and **VS14** (10 μM) induced the accumulation of yellow LC3II puncta in RFP-GFP-LC3 expressing HeLa cells. Scale bar = 10 μm.

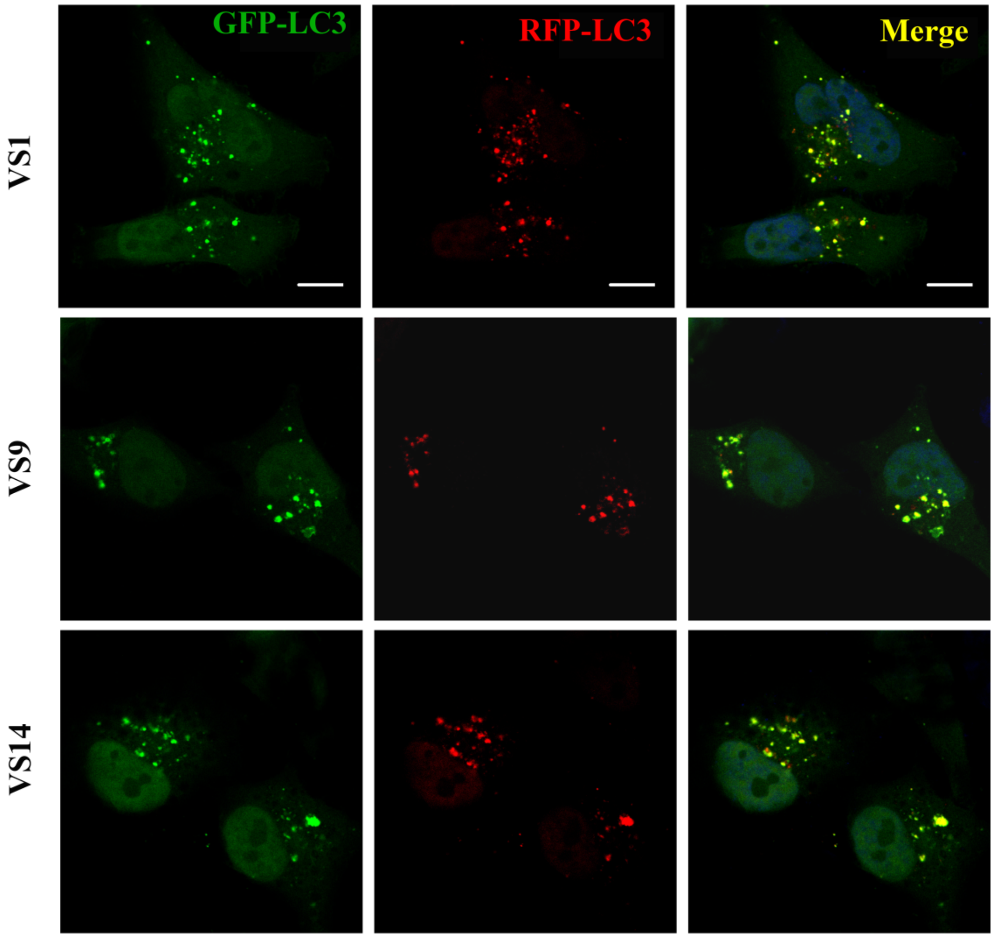

Figure S3. The effects of synthesized vacuolin-1 analogues on the accumulation of LC3-II in HeLa cells after 6 h treatment.

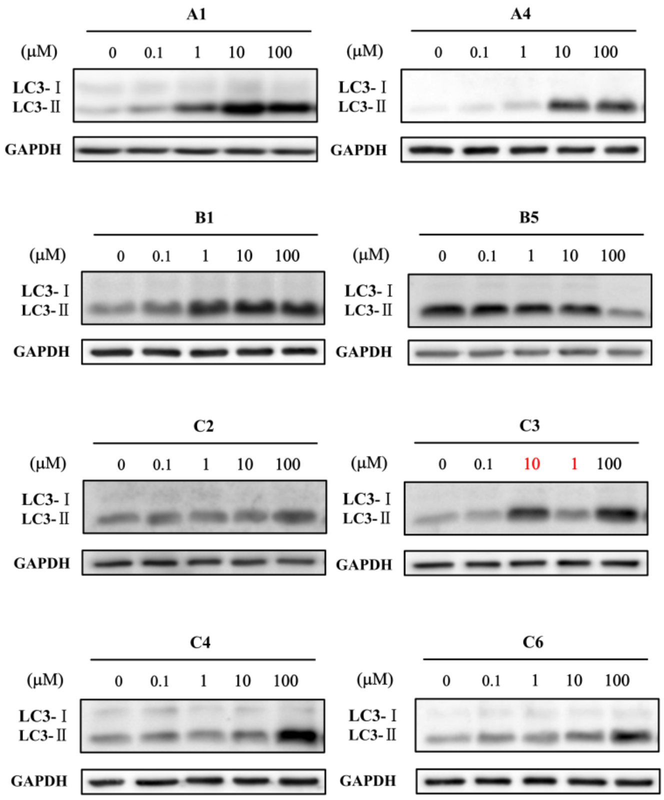

Figure S4. The effects of synthesized vacuolin-1 analogues on the accumulation of yellow LC3II puncta in RFP-GFP-LC3 expressing HeLa cells. Scale bar = 10 μm.

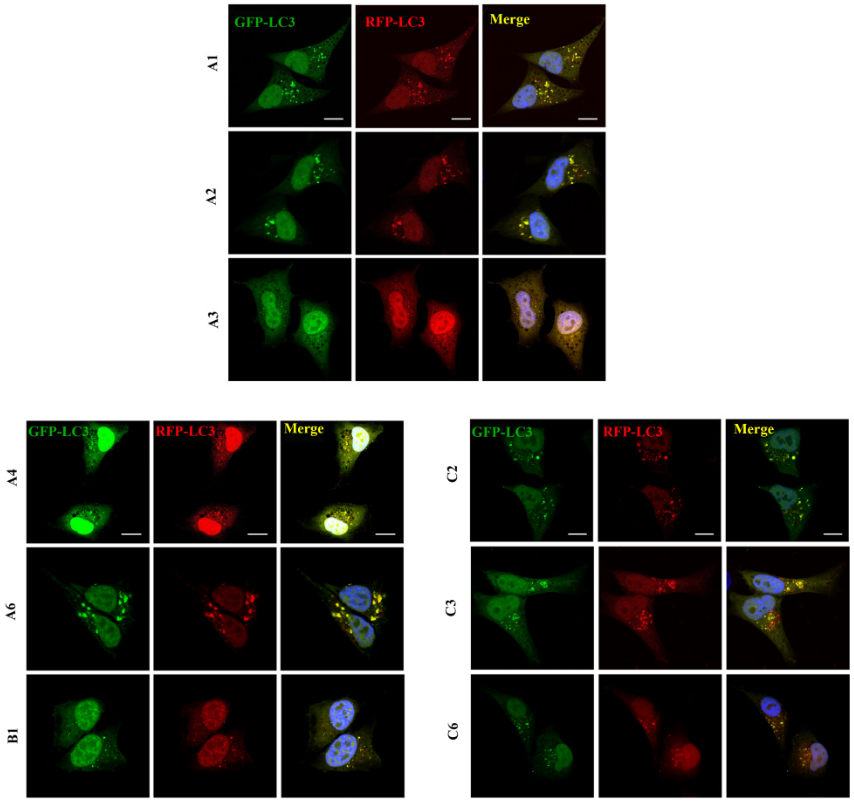

Quantification of Autophagy Inhibitor Western Blots

The ImageJ software was used to quantify the bands obtained by Western-blot. The grey values were determined in the selected band area and then normalized to the GAPDH loading control. The results are shown in **Figure S5, S6**

λ = (LC3-II Band – Background) / (GAPDH band – Background), λ_0: control group.

*, paired *t*-test, P<0.05 compared to control group.

Signal = λ/λ_0

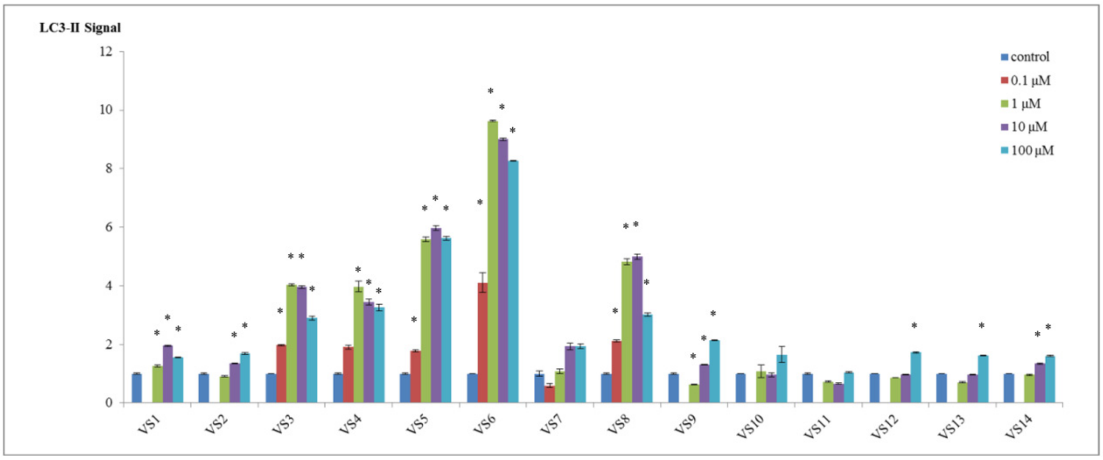

Figure S5 Compounds **VS1-VS9**.

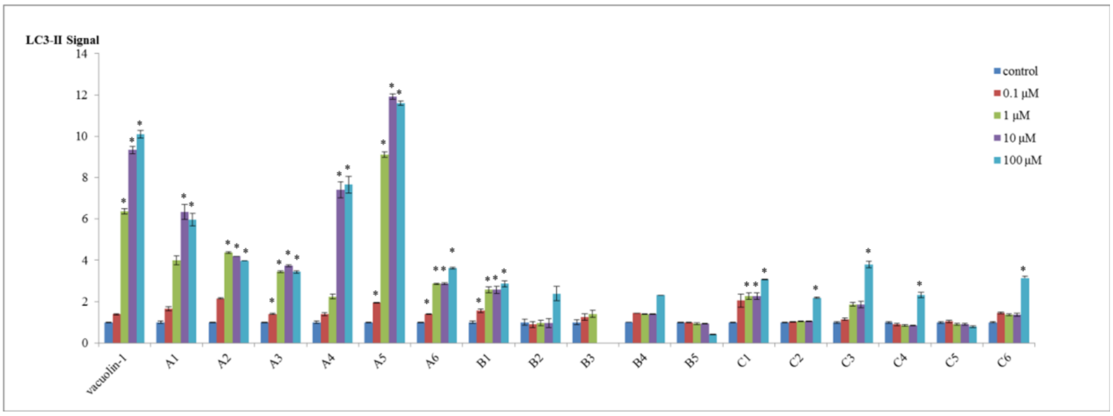

Figure S6 Compounds **A1-A5**, **B1-B5**, **C1-C6**.

^{1}H NMR spectra , ^{13}C NMR spectra and LC/ESI-TOF MS data

4,6-Dichloro-2-N,N-diphenylamino-1,3,5-triazine (**1a**)

Morpholine was used as amine and **1a** was prepared as light yellow solid. Yield 65%. ^{1}H NMR (400 MHz, $CDCl_3$) δ 7.46 – 7.39 (m, 4H), 7.36 – 7.30 (m, 2H), 7.30 – 7.27 (m, 4H). ^{13}C NMR (101 MHz, $CDCl_3$) δ 170.6, 165.9, 141.6, 129.5, 127.7, 127.2. MS (ESI-TOF$^+$) *m/z* 317 $[M+H]^+$.

4,6-Dichloro-2-(N-(3-methoxyphenyl)-N-phenylamino)-1,3,5-triazine (**1b**)

3-Methoxy-*N*-phenylaniline was prepared as described in the literature [1] and used as amine. **1b** was produced as white solid. Yield: 87%. ^{1}H NMR (400 MHz, $CDCl_3$) δ 7.43 (t, *J* = 7.4 Hz, 2H), 7.33 (dd, *J* = 15.1, 7.2 Hz, 4H), 6.89 (d, *J* = 7.6 Hz, 2H), 6.85 (s, 1H), 3.80 (s, 3H). ^{13}C NMR (101 MHz, $CDCl_3$) δ 170.6, 165.8, 160.4, 142.6, 141.5, 130.2, 129.5, 127.7, 127.1, 119.5, 113.5, 113.1, 55.5. (ESI-TOF$^+$) *m/z* 347 $[M+H]^+$.

4,6-Dichloro-2-(N-(naphthalen-1-yl)amino)-1,3,5-triazine **(1c)**

Light grew solid. Yield: 70%. ^{1}H NMR (400 MHz, $CDCl_3$) δ 7.98 – 7.88 (m, 3H), 7.87 (d, *J* = 8.4 Hz, 1H), 7.81 (d, *J* = 7.4 Hz, 1H), 7.63 – 7.54 (m, 3H). ^{13}C NMR (101 MHz, d_6-DMSO) δ 170.2, 169.2, 164.4, 135.0, 133.5, 131.0, 129.0, 128.0, 128.0, 127.2, 126.0, 121.9, 118.8. (ESI-TOF$^-$) *m/z* 289.1, 291.1 $[M-H]^-$

4,6-Dichloro-2-(N-(3-ethoxycarbonyl)phenylamino)-1,3,5-triazine **(1d)**

White solid. Yield: 89%. ^{1}H NMR (400 MHz, $CDCl_3$) δ 8.21 (s, 1H), 8.03 (s, 1H), 7.97 (d, *J* = 8.1 Hz, 1H), 7.92 (d, *J* = 7.7 Hz, 1H), 7.53 (t, *J* = 7.9 Hz, 1H), 4.46 (q, *J* = 7.1 Hz, 2H), 1.44 (t, *J* = 7.1 Hz, 3H). ^{13}C NMR (101 MHz, DMSO-d_6) δ 170.2, 169.3, 165.7, 164.3, 137.9, 131.0, 129.7, 126.2, 125.7, 122.2, 61.4, 14.6. (ESI-TOF$^-$) *m/z* 311 $[M-H]^-$.

4,6-Dichloro-2-(N-(hex-5-yn-1-yl)-N-phenylamino)-1,3,5-triazine **(1e)**

N-(Hex-5-yn-1-yl)aniline was prepared in the similar condition with *N*-Butylaniline described in the literature[2]. **1e** was prepared as light yellow solid. Yield:76 %. ^{1}H NMR (400 MHz, $CDCl_3$) δ 7.45 (t, *J* = 7.5 Hz, 2H), 7.36 (t, *J* = 7.4 Hz, 1H), 7.19 (d, *J* = 7.8 Hz, 2H), 4.00 (t, *J* = 7.4 Hz, 2H), 2.21 (t, *J* = 6.9 Hz, 2H), 1.93 (s, 1H), 1.80 – 1.69 (m, 2H), 1.60 – 1.50 (m, 2H). ^{13}C NMR (101 MHz, $CDCl_3$) δ 170.4, 170.1, 165.3, 140.5, 129.7, 128.1, 127.1, 83.7, 68.9, 50.7, 26.3, 25.3, 18.1. (ESI-TOF$^+$) *m/z* 321 $[M+H]^+$

4,6-Dichloro-2-(N-(4-ethoxy-4-oxobutyl)-N-phenylamino)-1,3,5-triazine **(1f)**

Ethyl 4-(phenylamino)butanoate was prepared in the similar condition with *N*-Butylaniline described in the literature[2]. **1f** was prepared as white solid. Yield:75 %. ^{1}H NMR (400 MHz, $CDCl_3$) δ 7.47 (t, *J* = 7.6 Hz, 2H), 7.38 (t, *J* = 7.4 Hz, 1H), 7.22 (d, *J* = 7.6 Hz, 2H), 4.13 (q, *J* = 7.1 Hz, 2H), 4.06 (t, *J* = 7.3 Hz, 2H), 2.38 (t, *J* = 7.3 Hz, 2H), 2.02 – 1.92 (m, 2H), 1.25 (t, *J* = 7.1 Hz, 3H). ^{13}C NMR (101 MHz, $CDCl_3$) δ 172.9, 170.8, 170.6, 165.8, 140.9, 130.1, 128.5, 127.4, 61.0, 50.9, 31.6, 23.0, 14.6. (ESI-TOF$^+$) *m/z* 355 $[M+H]^+$.

4,6-Dichloro-2-morpholino-1,3,5-triazine (**1g**)

White solid. Yield: 94%. ^{1}H NMR (400 MHz, $CDCl_3$) δ 3.97 – 3.86 (m, 4H), 3.81 – 3.71 (m, 4H). ^{13}C NMR (101 MHz, $CDCl_3$) δ 170.4, 164.1, 66.4, 44.5. (ESI-TOF$^+$) *m/z* 235 $[M+H]^+$.

6-Chloro-2-N,N-diphenylamino-4-morpholino-1,3,5-triazine (**2a**)

White solid. Yield 86%. ^{1}H NMR (400 MHz, $CDCl_3$) δ 7.37 (t, *J* = 7.7 Hz, 4H), 7.30 – 7.22 (m, 6H), 3.81 (s, 2H), 3.70 (s, 2H), 3.61 (s, 2H), 3.49 (s, 2H). ^{13}C NMR (101 MHz, $CDCl_3$) δ 169.9, 165.8, 164.4, 143.0, 128.9, 127.7, 126.3, 66.6, 66.5, 43.9, 43.6. (ESI-TOF$^+$) *m/z* 368 $[M+H]^+$.

6-Chloro-2-(N-(3-methoxyphenyl)-N-phenylamino)-4-morpholino-1,3,5-triazine (**2b***)*

White solid. Yield: 83%. ^{1}H NMR (400 MHz, $CDCl_3$) δ 7.37 (t, *J* = 7.4 Hz, 2H), 7.26 (dd, *J* = 18.6, 8.0 Hz, 4H), 6.87 (d, *J* = 12.5 Hz, 2H), 6.80 (d, *J* = 8.2 Hz, 1H), 3.81 (s, 2H), 3.78 (s, 3H), 3.70 (s, 2H), 3.61 (s, 2H), 3.51 (s, 2H). ^{13}C NMR (101 MHz, $CDCl_3$) δ 169.9, 165.7, 164.4, 160.0, 144.1, 143.0, 129.5, 128.9, 127.6, 126.4, 120.1, 113.7, 111.9, 66.6, 66.5, 55.4, 43.9, 43.7.

6-Chloro-4-morpholino-2- (*N-(naphthalen-1-yl)amino)-1,3,5-triazine* (**2c**)

White solid. Yield: 78%. ^{1}H NMR (400 MHz, $CDCl_3$) δ 8.03 – 7.94 (m, 1H), 7.93 – 7.85 (m, 2H), 7.75 (t, *J* = 7.5 Hz, 1H), 7.59 (br s, 1H), 7.53 (dt, *J* = 12.8, 6.0 Hz, 3H), 3.84 (s, 2H), 3.71 (s, 2H), 3.64 (s, 4H). ^{13}C NMR (101 MHz, $CDCl_3$) δ 169.7, 165.1, 164.6, 134.2, 132.5, 128.6, 127.93, 126.4, 126.2, 126.0, 125.4, 121.4, 121.4, 66.6, 44.0. (ESI-TOF$^+$) *m/z* 342 $[M+H]^+$.

6-Chloro-2-(N-(3-ethoxycarbonyl)phenylamino)-4-morpholino-1,3,5-triazine (**2d**)

White solid. Yield:85 %. ^{1}H NMR (400 MHz, $CDCl_3$) δ 8.43 (s, 1H), 7.81 (d, *J* = 7.6 Hz, 1H), 7.61 (d, *J* = 6.5 Hz, 1H), 7.43 (t, *J* = 7.9 Hz, 1H), 7.35 (s, 1H), 4.40 (q, *J* = 7.1 Hz, 2H), 3.96 – 3.86 (m, 4H), 3.83 – 3.71 (m, 4H), 1.41 (t, *J* = 7.1 Hz, 3H). ^{13}C NMR (101 MHz, d_6-DMSO) δ 168.9, 166.0, 164.5, 163.8, 139.6, 130.7, 129.4, 124.7, 124.0, 120.7, 66.2, 66.1, 61.2, 44.29, 44.2, 14.6. (ESI-TOF$^+$) *m/z* 364 $[M+H]^+$.

6-Chloro-2-(N-(4-ethoxy-4-oxobutyl)-N-phenylamino)-4-morpholino-1,3,5-triazine (**2f**)

White solid. Yield: 86%. ^{1}H NMR (400 MHz, $CDCl_3$) δ 7.41 (t, *J* = 7.6 Hz, 2H), 7.29 (dd, *J* = 9.0, 5.7 Hz, 1H), 7.23 (d, *J* = 7.6 Hz, 2H), 4.12 (q, *J* = 7.1 Hz, 2H), 4.01 (t, *J* = 6.9 Hz, 2H), 3.92 – 3.51 (m, 8H), 2.36 (t, *J* = 7.2 Hz, 2H), 2.01 – 1.89 (m, 2H), 1.24 (t, *J* = 7.1 Hz, 3H). ^{13}C NMR (101 MHz, $CDCl_3$) δ 173.0, 169.6, 169.2, 165.4, 142.0, 129.1, 127.6, 126.9, 66.7, 66.5, 60.5, 43.8, 31.4, 23.1, 14.2.

6-Chloro-2-(N-(4-cyanobutyl)-N-phenylamino)-4-morpholino-1,3,5-triazine (**2g**)

5-(Phenylamino)pentanenitrile was prepared similarly with *N*-butylaniline described in the literature [2]. **2g** was white solid. Yield: 42%. ^{1}H NMR (400 MHz, $CDCl_3$) δ 7.37 (t, *J* = 7.7 Hz, 2H), 7.27 – 7.21 (m, 3H), 4.00 (t, *J* = 6.7 Hz, 2H), 3.77 – 3.61 (m, 8H), 2.44 – 2.34 (m, 2H), 1.77 (dd, *J* = 14.1, 7.2 Hz, 2H), 1.70 (dd, *J* = 13.9, 6.9 Hz, 2H). ^{13}C NMR (101 MHz, $CDCl_3$) δ 168.6, 165.6, 165.0, 143.1, 128.8, 127.9, 126.2, 119.5, 66.8, 48.2, 43.6, 27.1, 22.7, 16.9. (ESI-TOF$^+$) *m/z* 373 $[M+H]^+$.

(S)-6-Chloro-2-N,N-diphenylamino-4-(3-methylmorpholino)-1,3,5-triazine (**2h**)

White solid. Yield 79%.^{1}H NMR (400 MHz, $CDCl_3$) δ 7.37 (t, *J* = 7.5 Hz, 4H), 7.32 – 7.21 (m, 6H), 4.79 – 4.29 (m, 1H), 4.25 – 3.78 (m, 2H), 3.76 – 3.51 (m, 2H), 3.51 – 3.33 (m, 1H), 3.32 – 3.03 (m, 1H), 1.25 (d, *J* = 53.1 Hz, 3H). ^{13}C NMR (101 MHz, $CDCl_3$) δ 169.9, 165.8, 164.1, 143.1, 128.9, 127.7, 126.3, 70.8, 66.7, 46.7, 38.9, 14.2. (ESI-TOF$^+$) *m/z* 382 $[M+H]^+$.

4-(4-(t-Butyloxycarbonyl)piperazin-1-yl)-6-chloro-2-N,N-diphenylamino-1,3,5-triazine (**2i**)

White solid. Yield 82%. ^{1}H NMR (400 MHz, $CDCl_3$) δ 7.37 (t, *J* = 7.7 Hz, 4H), 7.30 – 7.22 (m, 6H), 3.79 (s, 2H), 3.45 (s, 4H), 3.35 (s, 2H), 1.48 (s, 9H). ^{13}C NMR (101 MHz, $CDCl_3$) δ170.0, 165.8, 164.5, 154.7, 143.1, 129.0, 127.8, 126.4, 80.3, 43.3, 43.2, 28.5. (ESI-TOF$^+$) *m/z* 467 $[M+H]^+$.

6-Chloro-2-N,N-diphenylamino-4-((6-hydroxyhexyl)amino)-1,3,5-triazine (**2j**)

White solid showed only one spot by TLC, but appeared (by ^{13}C NMR) to be an inseparable mixture of isomers or tautomers, which was found in 2-(2-hydroxyethyl)amino-1,3,5-triazine derivatives [3] too. Yield 70%. ^{1}H NMR (400 MHz, DMSO-d_6) δ 8.09 – 7.87 (m, 1H), 7.38 (t, *J* = 7.4 Hz, 4H), 7.33 (d, *J* = 4.0 Hz, 4H), 7.25 (t, *J* = 6.3 Hz, 2H), 3.37 (t, *J* = 6.1 Hz, 2H), 2.92 (dd, *J* = 12.6, 6.2 Hz, 1H), 1.54 – 1.01 (m, 10H). ^{13}C NMR (101 MHz, DMSO-d_6) δ169.2, 168.6, 166.0, 165.9, 165.7, 165.5, 143.7, 143.4, 129.6, 129.3, 128.6, 128.4, 126.9, 126.9, 61.2, 33.0, 29.2, 29.0, 26.6, 25.6.

6-Chloro-2-N,N-diphenylamino-4-((2-hydroxyethyl)amino)-1,3,5-triazine (**2k**)

White solid. Yield 67%. ^{1}H NMR (400 MHz, DMSO-d_6) δ 7.88 (dt, *J* = 71.5, 5.6 Hz, 1H), 7.41 – 7.35 (m, 4H), 7.32 (d, *J* = 7.3 Hz, 4H), 7.24 (t, *J* = 7.1 Hz, 2H), 4.63 (dt, *J* = 40.0, 5.6 Hz, 1H), 3.46 (q, *J* = 6.1 Hz, 1H), 3.40 (s, 1H), 3.36 (dd, *J* = 11.5, 5.8 Hz, 1H), 3.29 (q, *J* = 6.0 Hz, 1H), 3.05 (q, *J* = 5.8 Hz, 1H). ^{13}C NMR (101 MHz, DMSO-d_6) δ 169.3, 168.6, 165.99, 165.96, 165.9, 165.7, 143.7, 143.4, 129.6, 129.4, 128.6, 128.4, 127.0, 126.9, 59.8, 59.5, 43.6, 43.5. (ESI-TOF$^+$) *m/z* 342 $[M+H]^+$.

Literatures:

1. Reddy Ch, V.; Kingston, J.V.; Verkade, J.G. (t-bu)2pn=p(i-bunch2ch2)3n: New efficient ligand for palladium-catalyzed c-n couplings of aryl and heteroaryl bromides and chlorides and for vinyl bromides at room temperature. *J. Org. Chem.* **2008**, *73*, 3047-3062.
2. Castillo, J.C.; Orrego-Hernandez, J.; Portilla, J. Cs2co3-promoted direct n-alkylation: Highly chemoselective synthesis of n-alkylated benzylamines and anilines. *Eur. J. Org. Chem.* **2016**, 3824-3835.
3. Meyer, A.H.; Dybala-Defratyka, A.; Alaimo, P.J.; Geronimo, I.; Sanchez, A.D.; Cramer, C.J.; Elsner, M. Cytochrome p450-catalyzed dealkylation of atrazine by rhodococcus sp strain n186/21 involves hydrogen atom transfer rather than single electron transfer. *Dalton Transactions* **2014**, *43*, 12175-12186.
